# The impact of driving pressure on postoperative pulmonary complication in patients with different respiratory spirometry

**DOI:** 10.1038/s41598-022-24627-2

**Published:** 2022-12-03

**Authors:** Eun Jung Oh, Bo-Guen Kim, Sukhee Park, Sangbin Han, Beomsu Shin, Hyun Lee, Sun Hye Shin, Jeayoun Kim, Dancheong Choi, Eun Ah Choi, Hye Yun Park

**Affiliations:** 1 Department of Anesthesiology and Pain Medicine, Samsung Medical Center, Sungkyunkwan University School of Medicine, Seoul, Korea; 2 Division of Pulmonary and Critical Care Medicine, Department of Medicine, Samsung Medical Center, Sungkyunkwan University School of Medicine, Seoul, Korea; 3 Department of Anesthesiology and Pain Medicine, International St. Mary’s Hospital, Catholic Kwandong University School of Medicine, Incheon, Korea; 4 Department of Medicine, Yonsei University Wonju College of Medicine, Wonju, Korea; 5 Department of Internal Medicine, Hanyang Medical Center, Hanyang University College of Medicine, Seoul, Korea

**Keywords:** Outcomes research, Risk factors

## Abstract

Risk factors for postoperative pulmonary complication (PPC) have not been determined according to preoperative respiratory spirometry. Thus, we aimed to find contributors for PPC in patients with restrictive or normal spirometric pattern. We analyzed 654 patients (379 with normal and 275 with restrictive spirometric pattern). PPCs comprised respiratory failure, pleural effusion, atelectasis, respiratory infection, and bronchospasm. We analyzed the association between perioperative factors and PPC using binary logistic regression. In particular, we conducted subgroup analysis on the patients stratified according to preoperative spirometry. Of 654 patients, 27/379 patients (7.1%) with normal spirometric pattern and 33/275 patients (12.0%) with restrictive spirometric pattern developed PPCs. Multivariable analysis demonstrated that high driving pressure was the only intraoperative modifiable factor increasing PPC risk (OR = 1.13 [1.02–1.25], p = 0.025). In the subgroup of patients with restrictive spirometric pattern, intraoperative driving pressure was significantly associated with PPC (OR = 1.21 [1.05–1.39], p = 0.009), whereas driving pressure was not associated with PPC in patients with normal spirometric pattern (OR = 1.04 [0.89–1.21], p = 0.639). In patients with restrictive spirometric pattern, greater intraoperative driving pressure is significantly associated with increased PPC risk. In contrast, intraoperative driving pressure is not associated with PPC in patients with normal spirometric pattern.

## Introduction

The incidence of postoperative pulmonary complication (PPC) is as high as 23% and PPC is related to prolonged hospital stay and mortality^1,2^. Thus, many previous studies gave efforts to determine modifiable contributing factors for PPC to prevent PPC^1^. Those efforts may be more important for patients with suboptimal lung functions. In this regard, obstructive lung disease like chronic obstructive pulmonary disease (COPD), which is known to increase the risk of PPC and postoperative mortality^3,4^, has been being widely studied with respect to the association with PPC^3–6^.

Of importance, PPC incidence of patients without obstructive lung disease has been reported as high as PPC incidence of patients with obstructive lung disease^2,7^.

A previous study demonstrated that PPC risk was 4.2 times greater in patients with moderate to severe restrictive spirometric pattern compared to patients with normal spirometric pattern^8^. Moreover, PPC incidence of patients with normal spirometric pattern was reported to reach 20% in previous studies^9,10^. Thus, it is important to find modifiable contributing factors for PPC in patients with restrictive or normal spirometric pattern, which has been not actively performed compared to patients with obstructive spirometric pattern^11^.

We hypothesized that risk factors for PPC differ according to patients’ baseline lung physiology, demonstrated by preoperative spirometry. As an extension of our previous study of patients with COPD^12^, this study aimed to determine intraoperative factors related to PPCs in patients without obstructive spirometric pattern. For this purpose, we included patients with restrictive spirometric pattern or normal spirometric pattern.

## Methods

### Subjects and data sources

**Figure 1:**
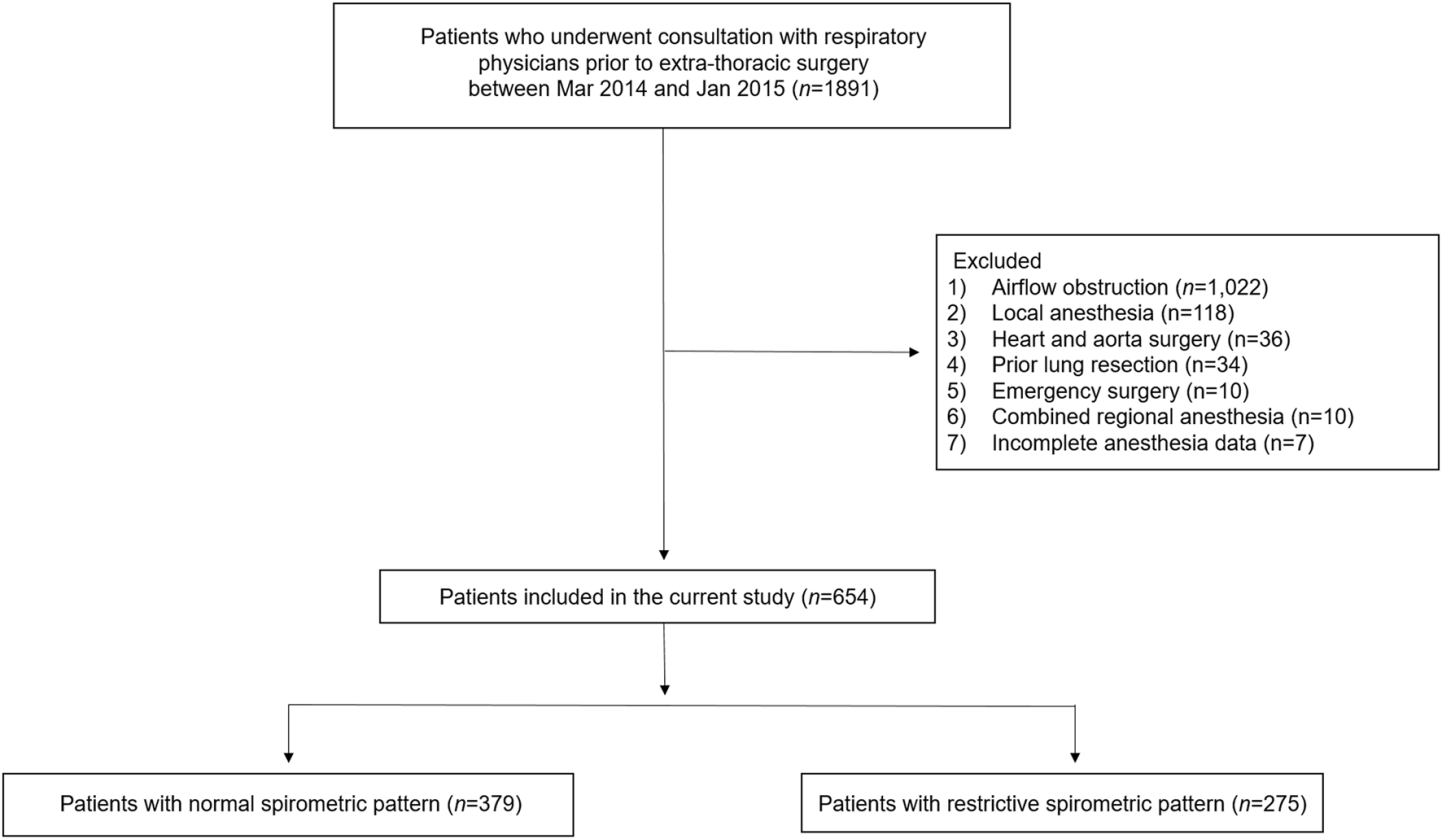
Consolidated standards of reporting trials diagram.

We initially screened 1891 adults consulted to the respiratory physician prior to extra-pulmonary surgery between March 2014 and January 2015. All consulted patients were registered in our prospectively collected institutional PPC database^13^. The consultation criteria were patients with underlying lung disease, past history of lung disease, such as pulmonary infection, abnormal arterial partial pressure of oxygen (PaO_2_) in arterial blood gas analysis, older age (> 60 years), and the attending anesthesiologist’s requirement. We excluded 1022 patients with obstructive lung disease (i.e., pre-bronchodilator FEV_1_/FVC < 0.7)^12^ or who showed abnormal pulmonary findings in preoperative examination when abnormal pulmonary findings were defined as pulmonary radiologic findings of atelectasis or pleural effusion in preoperative examination, pulmonary infection within 1 month, and PaO_2_ < 60 mmHg. We further excluded 118 patients who underwent local anesthesia, 10 patients with combined regional nerve block, 36 patients who underwent heart or aorta surgery, and 34 patients with previous history of lung resection. Also, we excluded 10 patients who underwent emergent surgery, and 7 patients with incomplete intraoperative data. Finally, the remaining 654 patients of non-obstructive spirometric pattern (379 with normal spirometric pattern and 275 with restrictive spirometric pattern) were included in the current study (Fig. 1). Restrictive spirometric pattern was defined based on the spirometry without lung volume measurement. Thus, restrictive spirometric pattern refers to a reduction in vital capacity, but is not the same with the diagnosis of restrictive disease. The study protocol was approved by the institutional review board of Samsung Medical Center on 19 February 2020 (SMC 2020-01-048-003) and written informed consent was waived due to the retrospective nature by the institutional review board of Samsung Medical Center. All methods were performed in accordance with the relevant guidelines and regulations.

### Definition of normal and restrictive spirometric pattern

Preoperative spirometry was performed using a Vmax 22 apparatus (SensorMedics, Yorba Linda, CA, USA) according to American Thoracic Society/European Respiratory Society criteria^14^. Spirometry was generally performed within 1 month before surgery. Absolute values of forced expiratory volume in one second (FEV_1_) and forced vital capacity (FVC) were obtained, and percentage of the predicted values for FEV_1_ and FVC were calculated using a reference equation obtained in a representative Korean sample^15^. Normal spirometric pattern was defined when there was no airflow obstruction (FEV_1_/FVC ≥ 0.7) and FVC was ≥ 80% of the predicted value. Restrictive spirometric pattern was defined when FEV_1_/FVC was ≥ 0.7 and FVC was < 80% of the predicted value. The severity of restrictive spirometric pattern was classified as mild (FVC ranges from 60 to 79% of the predicted value) and moderate-to-severe restrictive (FVC < 60% of the predicted value), as described previously^16^.

### PPC evaluation

PPC was evaluated daily for 7 postoperative days and categorized as respiratory failure, respiratory infection, pleural effusion, atelectasis, and bronchospasm (Supplementary Table S1)^2,12,13^. Two respiratory physicians (B. Shin and H. Lee) reviewed the electronic medical records, laboratory, and radiologic findings and confirmed whether the patients fulfilled the definitions of PPCs and recorded the results in the PPC database. All preoperative and postoperative data analyzed in the current study (including PPC, postoperative hospital stay, and 30-/90-day mortality) were already collected in the PPC database irrespective of the study.

### Variables

Intraoperative data regarding intubation difficulty, anesthetic agents, mechanical ventilation parameters at the end of surgery, hemodynamics, fluid infusion, blood loss, core temperature, anesthesia duration, and neuromuscular blockade/reversal were newly collected from electronic medical records. Among the mechanical ventilation parameters, static lung compliance was defined as tidal volume divided by driving pressure and driving pressure was defined as plateau pressure minus positive end-expiratory pressure (PEEP).

### Statistical analysis

The primary outcome was PPC. We evaluated the association between perioperative variables and PPC using binary logistic regression in the whole cohort as well as in the subgroups stratified by preoperative spirometric pattern (normal spirometry or restrictive spirometry) and the results are described as odds ratio (OR) with 95% confidence interval. The final multivariable model for the whole cohort was generated using the entry variable selection method with independent variables with p < 0.2 during univariable analysis being included^17^. Static compliance, plateau pressure, and PEEP were not included in the multivariable analysis due to mathematical connection to driving pressure. Multi-collinearity was checked using the variance inflation factor. Low-tidal-volume ventilation was defined when tidal volume was < 8 mL per kilogram of ideal body weight based on previous research^18,19^. We modified albumin as a binary variable with the cutoff value of 3.5 g/dL, which is known to be related to surgical prognosis^20^. Also, we converted age and hemoglobin to binary variables with the cut-off values obtained by using ROC analysis. The subgroup analysis (of patients with normal spirometry or restrictive spirometry) was conducted by multivariable logistic regression with the three variables confirmed to be significant during the multivariable analysis for the whole cohort being included (age, laparotomy, and driving pressure). Continuous variables such as statistic compliance, driving pressure, and plateau pressure are summarized as median (25th, 75th percentile) and compared using *t* test or Wilcoxon rank-sum test, while categorical variables are presented as frequency (%) and compared using chi-square test or Fisher’s exact test. All reported p values were two-sided and p < 0.05 was considered statistically significant. Analysis were performed using SPSS 26.0 (IBM Corp., Chicago, IL, USA).

## Results

### Incidence of PPC

Of the total 654 patients, 60 patients (9.2%) developed 114 PPCs while 44 patients had multiple PPCs: 27 of 379 patients (7.1%) with normal spirometric pattern and 33 of 275 patients (12.0%) with restrictive spirometric pattern developed PPCs (OR = 1.78[1.04–3.03], p = 0.035). In particular, 11 of 164 patients (6.7%) with mild restrictive spirometric pattern and 22 of 112 patients (19.6%) with moderate-to-severe restrictive spirometric pattern developed PPCs (OR = 3.37 [1.57–7.29], p = 0.002). The proportion of each PPC among 68 PPCs in patients with restrictive spirometric pattern was as follows: respiratory failure (41.2%), atelectasis (26.5%), pleural effusion (22.1%), and respiratory infection (10.3%). The proportion of each PPC among 46 PPCs in patients with normal spirometric pattern was as follows: pleural effusion (41.3%), atelectasis (34.8%), respiratory failure (19.6%), and respiratory infection (4.3%).

### Analysis for the whole study cohort

As shown in Table 1, the results of univariable analysis demonstrated that driving pressure was the only significant intraoperative modifiable factor contributing to PPC risk (11.5 [10.0–14.0] cmH_2_O in patients without PPCs vs. 13.0 [11.0–14.5] cmH_2_O in patients with PPCs, p = 0.026). Multivariable analysis confirmed the significance of driving pressure (OR = 1.11 [1.00–1.23], p = 0.041, Table 2). In addition to greater driving pressure, older age (OR = 2.29 [1.27–4.13, p = 0.006) and open abdominal surgery (OR = 2.93 [1.54–5.54], p = 0.001) were determined as independent risk factors for PPC.

**Table 1:** Perioperative variables of patients with or without postoperative pulmonary complications (PPCs) in the whole study cohort.

	Descriptive statistics		Univariable analysis	
	Without PPCs (n = 594)	With PPCs (n = 60)	OR (95% CI)	p-value
**Patient factors**				
Age > 70 years	224 (37.7)	36 (60.0)	2.48 (1.44–4.26)	0.001
Male sex	292 (49.2)	27(45.0)	1.18 (0.69–2.02)	0.539
Body mass index (kg/m^2^)	24.1 (21.8–26.2)	23.3 (20.5–25.7)	0.97 (0.91–1.05)	0.455
Hemoglobin > 10 g/dL	53 (8.9)	10 (16.7)	2.04 (0.98–4.26)	0.057
Albumin < 3.5 g/dL	59 (9.9)	11 (18.3)	2.04 (1.00–4.13)	0.049
Neutrophil-to-lymphocyte ratio	2.0 (1.4–3.1)	2.0 (1.3–3.3)	1.05 (1.01–1.09)	0.023
ASA class ≥ 2	443 (74.6)	50 (83.3)	1.70 (0.84–3.44)	0.138
Ever smoker	181 (30.5)	14 (23.3)	0.69 (0.37–1.30)	0.252
Restrictive spirometry severity				< 0.001
Normal	351 (59.1)	27 (45.0)	Reference	
Mild	153 (25.8)	11 (18.3)	0.94 (0.45–1.93)	
Moderate to severe	90 (15.2)	22 (36.7)	3.18 (1.73–5.84)	
Comorbidity				
Diabetes	142 (23.9)	11 (18.3)	0.72 (0.36–1.41)	0.333
Hypertension	244 (41.1)	29 (48.3)	1.34 (0.79–2.28)	0.279
Congestive heart failure	50 (8.4)	8 (13.3)	1.67 (0.75–3.72)	0.206
Malignancy	268 (45.1)	36 (60.0)	1.83 (1.06–3.14)	0.029
Interstitial lung disease	27 (4.5)	1 (1.7)	0.36 (0.05–2.67)	0.315
**Procedure factors**				
Type of surgery				< 0.001
Abdominal	91 (15.3)	22 (36.7)	Reference	
Laparoscopic or robotic	102 (17.2)	11 (18.3)	0.44 (0.21–0.96)	
Neurosurgery	141 (23.7)	7 (11.7)	0.14 (0.05–0.37)	
Head and neck	46 (7.7)	3 (5.0)	0.25 (0.07–0.87)	
Others*	214 (36.0)	17 (28.3)	0.30 (0.15–0.58)	
**Intraoperative factors**				
Laryngeal mask airway use	12 (2.0)	0 (0)	–	–
Anesthetic maintenance agent				0.108
Sevoflurane	310 (52.2)	29 (48.3)	Reference	
Isoflurane	17 (2.9)	4 (6.7)	2.52 (0.79–7.97)	
Desflurane	80 (13.5)	13 (21.7)	1.74 (0.86–3.49)	
Propofol	187 (31.5)	14 (23.3)	0.80 (0.41–1.55)	
Neuromuscular blocking agent				0.615
Rocuronium	461 (77.9)	50 (83.3)	Reference	
Vecuronium	95 (16.0)	7 (11.7)	0.68 (0.30–1.54)	
Cisatracurium	36 (6.1)	3 (5.0)	0.77 (0.23–2.59)	
Mechanical ventilation parameters at the end of surgery				
Static compliance (mL/cmH_2_O)	38.8 (31.4–48.0)	34.7 (28.9–46.7)	0.98 (0.95–1.00)	0.050
Driving pressure (cmH_2_O)	11.5 (10.0–14.0)	13.0 (11.0–14.5)	1.11 (1.01–1.21)	0.026
Low-tidal-volume ventilation	294 (49.5)	24 (40.0)	1.47 (0.86–2.53)	0.163
Plateau pressure (cmH_2_O)	13 (12–15)	15 (12–16)	1.07 (0.98–1.16)	0.116
PEEP ≥ 5 cmH_2_O	79 (13.3)	5 (8.3)	0.59 (0.23–1.53)	0.278
Crystalloid infusion (mL/kg/h)	5.9 (4.2–8.3)	7.0 (4.3–11.2)	1.01 (0.97–1.04)	0.707
Colloid infusion	248 (41.8)	24 (40.0)	0.93 (0.54–1.60)	0.793
Red blood cell transfusion	71 (12.0)	9 (15.0)	1.30 (0.61–2.75)	0.493
Estimated blood loss (mL)	150 (50–300)	150 (63–300)	1.00 (1.00–1.00)	0.183
MBP < 60 mmHg for > 30 min	66 (11.1)	9 (15.0)	1.41 (0.67–3.00)	0.370
Hypothermia	240 (40.6)	29 (48.3)	1.37 (0.80–2.33)	0.248
Sugammadex neuromuscular reversal	54 (9.1)	4 (6.7)	0.71 (0.25–2.05)	0.531
Anesthesia duration (minutes)	193 (140–283)	196 (144–319)	1.00 (1.00–1.00)	0.552

Data are presented as median (25th percentile, 75th percentile) or frequency (%). *ASA* American society of anesthesiologist, *MBP* mean blood pressure, PEEP positive end-expiratory pressure. *Others included breast, endocrine, vascular, orthopedic and spinal, gynecologic, urologic, ophthalmologic, and plastic surgery.

**Table 2:** Multivariable analysis for the whole study cohort and the subgroups stratified by preoperative spirometry.

	Whole study cohort (n = 654)		Normal spirometric pattern (n = 379)		Restrictive spirometric pattern (n = 275)	
	OR (95% CI)	p-value	OR (95% CI)	p-value	OR (95% CI)	p-value
**Non-modifiable factor**						
Age > 70 years	2.29 (1.27–4.13)	0.006	1.31 (058–2.95)	0.521	4.42 (1.82–10.70)	0.001
Hemoglobin > 10 g/dL	1.23 (0.51–2.97)	0.641				
Albumin < 3.5 g/dL	0.84 (0.34–2.11)	0.714				
Neutrophil-to-lymphocyte ratio	1.03 (0.98–1.08)	0.309				
ASA class ≥ 2	1.27 (0.59–2.74)	0.539				
Restrictive severity		0.054				
Normal	Reference					
Mild	0.67 (0.31–1.49)					
Moderate to severe	1.88 (0.90–3.96)					
Malignancy	1.73 (0.95–3.13)	0.071				
Open abdominal surgery	2.93 (1.54–5.54)	0.001	2.33 (0.94–5.79)	0.068	6.40 (2.82–14.54)	< 0.001
**Modifiable factor**						
Anesthetic maintenance agent		0.147				
Sevoflurane	Reference					
Isoflurane	1.98 (0.55–7.07)					
Desflurane	2.13 (1.01–4.48)					
Propofol	0.93 (0.45–1.92)					
Driving pressure	1.11 (1.00–1.23)	0.041	1.04 (0.89–1.20)	0.640	1.19 (1.04–1.37)	0.014
Low-tidal-volume ventilation	1.56 (0.86–2.83)	0.142				
Estimated blood loss (mL)	1.00	0.664				

Multivariable analysis was performed using the entry method including 12 variables with p of < 0.2 in univariate analysis. Static compliance was not included in the multivariable analysis process due to mathematical connection to the driving pressure. Subgroup analysis was performed by logistic regression including the three co-variables confirmed to be significant in the final multivariable model for the whole study cohort (age, open abdominal surgery, and driving pressure). Type of surgery was included in the multivariable analysis as a dichotomous variable (open abdominal surgery or other surgeries).

As shown in Table 3, the length of postoperative hospital stay (9 [6–13] days vs. 10 [7–21] days, p = 0.115) and the 30-day mortality (1.2% vs. 3.3%, p = 0.193) were insignificantly greater in patients with PPCs than in patients without PPCs, whereas 90-day mortality was significantly greater in patients with PPCs than in patients without PPCs (13.3% vs. 3.4%, p = 0.001).

**Table 3:** Association between postoperative pulmonary complication (PPC) and postoperative clinical courses.

	Without PPCs	With PPCs	p-value
**The whole study cohort (n = 654)**			
Length of postoperative hospital stay (days)	9 (6–13)	10 (7–21)	0.115
30-Day mortality	7 (1.2)	2 (3.3)	0.193
90-Day mortality	20 (3.4)	8 (13.3)	0.001
**Patient with normal spirometric pattern (n = 379)**			
Length of postoperative hospital stay (days)	9 (6–11)	7 (6–10)	0.203
30-Day mortality	1 (0.3)	0	–
90-Day mortality	5 (1.4)	1 (3.7)	0.380
**Patient with restrictive spirometric pattern (n = 275)**			
Length of postoperative hospital stay (days)	10 (6–16)	18 (10–28)	0.086
30-Day mortality	6 (2.5)	2 (6.1)	0.247
90-Day mortality	15 (6.2)	7 (21.2)	0.005

Data are presented as median (25th percentile, 75th percentile) or frequency (%).

### Subgroup analysis for patients with normal spirometry

**Figure 2:**
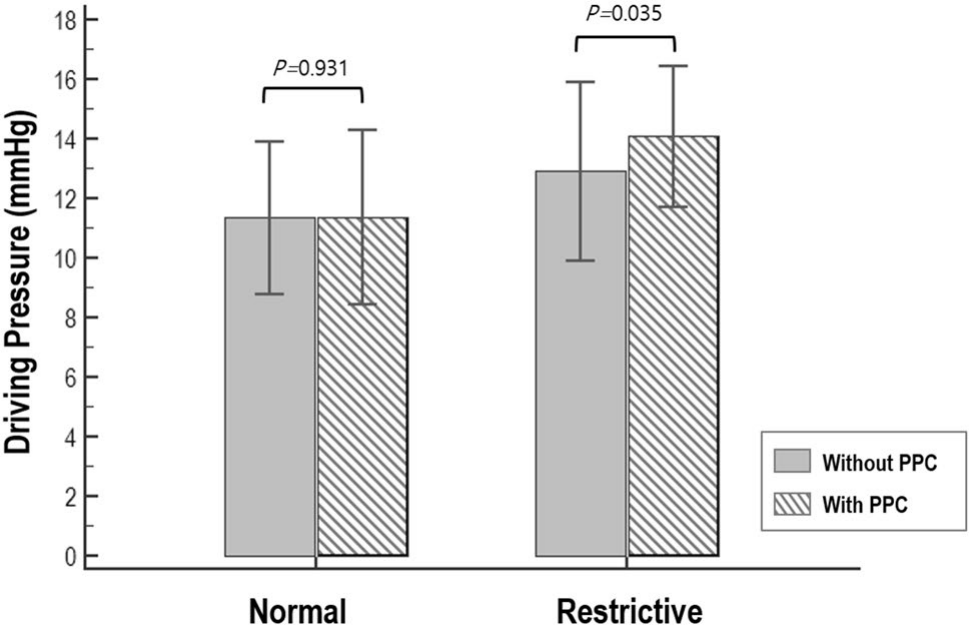
The association between intraoperative driving pressure and postoperative pulmonary complication (PPC) in the subgroup of patients with normal preoperative spirometry and in the subgroup of patients with restrictive preoperative spirometry.

In the subgroup of patients with normal spirometric pattern, driving pressure was not associated with PPC after adjusting for age and laparotomy (OR = 1.04 [0.89–1.20], p = 0.640, Table 2). As shown in Fig. 2, driving pressure was not different between patients without PPCs and patients with PPCs (11.0 [9.5–13.0] cmH_2_O vs. 11.0 [9.0–13.5] cmH_2_O, p = 0.931). The length of postoperative hospital stay and the risk of 30-/90-day mortality were not significantly different between patients without PPCs and patients with PPCs (Table 3).

### Subgroup analysis for patients with restrictive spirometry

In the subgroup of patients with restrictive spirometric pattern, driving pressure positively correlated with PPC risk after adjusting for age and laparotomy (13.5 [12.3–15.0] cmH_2_O in patients with PPCs and 12.5 [11.0–15.0] cmH_2_O in patients without PPCs, OR = 1.19 [1.04–1.37], p = 0.014, Table 2 and Fig. 2). The length of postoperative hospital stay and 30-day mortality were insignificantly greater in patients with PPCs (p = 0.138 and p = 0.267, respectively, Table 3), whereas 90-day mortality was significantly greater in patients with PPCs (21.2% vs. 6.2%, OR = 4.07 [1.52–10.91], p = 0.005). The length of postoperative hospital stay (10 [6–16] days vs. 18 [10–28] days, p = 0.086) and the 30-day mortality (2.5% vs. 6.1%, p = 0.247) were insignificantly greater in patients with PPCs than in patients without PPCs, whereas 90-day mortality was significantly greater in patients with PPCs than in patients without PPCs (21.2% vs. 6.2%, p = 0.005).

## Discussion

This study aimed to find modifiable anesthetic factors contributing to the development of PPC in patients with normal or restrictive spirometric pattern. To our knowledge, there have been no studies evaluating the impact of driving pressure according to different lung physiologies. Two pulmonary physicians independently evaluated the presence and kind of PPCs irrespective of the study and recorded the results in an institutional PPC database. First, we found that driving pressure was the only modifiable intraoperative factor affecting PPC risk. Of importance, greater driving pressure was significantly associated with increased PPC risk only in patients with restrictive spirometric pattern but not in patients with normal spirometric pattern. In combination with our previous study^12^ demonstrating that driving pressure was not significantly associated with PPC risk in patients with obstructive lung disease, the current study suggests that driving pressure has a particular impact on the development of PPC in patients with decreased lung compliance. Second, we found that PPC risk is two-fold greater in patients with restrictive spirometric pattern than in patients with normal spirometric pattern. Among patients with restrictive spirometric pattern, the majority of PPC occurred in patients with moderate-to-severe restrictive pattern (19.6%) rather than in patients with mild restrictive pattern (6.7%). Third, the kind of PPC was more severe in patients with restrictive spirometric pattern. The most common PPC was respiratory failure in patients with restrictive spirometric pattern while it was pleural effusion in patients with normal spirometric pattern, recommending particular efforts for patients showing restrictive spirometric pattern before surgery.

Driving pressure is considered as a surrogate parameter representing transpulmonary pressure^21^. Transpulmonary pressure consists of the pressure to overcome the inward elastic recoil of the lung (intrapleural pressure) and the pressure through the airway (airway opening pressure). Accordingly, when there is no airway flow (i.e. zero flow), transpulmoanry pressure approximates the lung elastic recoil components, which is the pressure to quantify lung stress and lung injury risk during mechanical ventilation^21,22^. Because, measuring the exact transpulmonary pressure requires additional equipment and training, recent studies used driving pressure to estimate transpulmonary pressure and demonstrated its association with PPC^23^.

In specific situations, driving pressure represents the pressure from the whole respiratory system including the pressure generated out of the lung, such as chest wall, in addition to transpulmonary pressure^24^. During mechanical ventilation, driving pressure can be interpreted as the pressure applied to the entire respiratory system to achieve the tidal volume; thus, the lowest driving pressure is achieved when the ventilation is performed based on patients' functional lung size^23^. Functional lung size is defined as the ideal lung volume for aeration during mechanical ventilation without over-distension or under-ventilation^25^. Optimizing the tidal volume to the functional lung size leads to a minimum driving pressure and maximum lung compliance.

In our study, low driving pressure showed significant protective effect in patients with restrictive spirometric pattern. This finding is in consistent with previous studies of patients with acute respiratory distress syndrome (ARDS) with decreased lung size and low compliance as patients with restrictive spirometric pattern. In ARDS patients, driving pressure was significantly associated with PPC risk and mortality risk^26,27^. In a previous study of patients undergoing thoracic surgery, individually adjusted driving pressure-guided ventilation decreased driving pressure compared to the conventional protective ventilation (9 [8–10] cmH2O vs. 10 [9–11] cmH2O, p < 0.001), while the driving pressure-guided ventilation decreased PPC incidence by 16%^28^. The finding that even small decrease in driving pressure positively affects clinical outcome is in line with the current study: the median difference in driving pressure between patients without PPCs and with PPCs was 1.2 cmH_2_O in the subgroup of patients with restrictive spirometric pattern while PPC risk increased 19% for each 1.0 cmH_2_O increase in driving pressure. The previous and current studies suggest the importance to give efforts to make small differences in driving pressure for patients with restrictive spirometric pattern, the so-called driving pressure-guided ventilation.

Restrictive spirometric pattern may reflect a number of diseases (e.g. interstitial lung disease, diabetes, obesity, cardiovascular disease, hypertension, etc.)^29^. These underlying diseases are thought to cause restrictive spirometric pattern in the current study are shown in Table 1. Regardless of the cause of restrictive spirometric pattern, overall lung volume reduction in patients with restrictive spirometric pattern decreases the lung compliance and increases the work of breathing compared to normal lung^30,31^. Thus, transpulmonary pressure increases in patients with restrictive spirometric pattern and it may be more important to minimize the pressure applying to the lung by adjusting tidal volume and PEEP during mechanical ventilation.

In contrast to the patients with restrictive spirometric pattern, driving pressure was not associated with PPC risk in patients with normal spirometric pattern. Previous meta-analysis^23^, which included 17 randomized controlled studies of abdominal, thoracic, and cardiac surgery, demonstrated the significant association between driving pressure and PPC risk. However, in this meta-analysis, only 6 of 17 studies took into account preoperative spirometry and some studies did not limit the subjects to patients without lung disease or abnormal spirometry. None of the studies included in the meta-analysis analyzed as large number of subjects with normal spirometric pattern as the current study. Our study has additional strength because 258 of 379 patients (68.1%) with normal spirometric pattern underwent extra-thoracic and extra-abdominal surgery, while the meta-analysis included only 24 patients (1.1%) with extra-thoracic and extra-abdominal surgery^23^. When the surgical field is close to the thorax as in thoracic or abdominal surgery, the airway pressure could differ dynamically depending on the surgical procedure. Thus, it is difficult to actually estimate the pressure generated from the external lung and assume driving pressure as a surrogate parameter of transpulmonary pressure^32^. Because the majority surgical fields were not close to the thorax in the current study, driving pressure was thought to better represent transpulmonary pressure in a better environment to evaluate the real association between driving pressure and PPC risk.

Low driving pressure did not show protective effects in our previous study of patients with COPD^12^, being in contrast to patients with restrictive spirometric pattern in the current study. The difference may be attributable to their different lung physiology. The physiological basis of COPD is decreased elasticity of the lung and airflow limitation resulting in lung hyperinflation^33^. In the hyperinflated lung, the compliance is increased resulting in a large divergence between driving pressure and transpulmonary pressure^34^. However, in patients with restrictive lung disease and reduced lung compliance, the majority fraction of driving pressure is used to inflate the lung, reducing the difference between driving pressure and transpulmonary pressure. Thus, driving pressure can be a reasonable surrogate parameter representing transpulmonary pressure in patients with restrictive spirometric pattern, regardless of the abnormal lung compliance, while this approach may not be relevant for patients with obstructive lung disease. In this regard, the difference between driving pressure and transpulmonary pressure in patients with COPD may influenced the insignificance of driving pressure. Future research is needed to evaluate the association between transpulmonary pressure and PPC risk or the impact of driving pressure-guided ventilation on PPC in patients with different lung physiologies or spirometric patterns.

This study has some limitations. First, there was a potential risk of selection bias because patients who did not perform preoperative pulmonary function test prior to extra-pulmonary surgery were not included in the study. In this regard, young patients without previous lung abnormalities were not included in the study. However, PPC risk is known to increase with age and the PPC incidence increases markedly at 80 years old^2^. Analyzing patients at higher risk for PPC better suits the purpose of the study. Second, as this was a retrospective study, we were unable to provide transpulmonary pressure and verify the relationship between driving pressure and transpulmonary pressure. Based on the recent data, end-expiratory esophageal balloon pressures can reliably estimate transpulmonary pressures^35^. Thus, future studies simultaneously measuring transpulmonary pressure (or end-expiratory esophageal balloon pressure) and driving pressure are warranted to confirm the relationship between driving pressure and transpulmonary pressure according to different lung physiology or spirometric pattern. Third, preoperative PPC risk evaluation was performed based on a guideline and lung volume was not measured in general because lung volume measurement is time consuming and expensive^13,14^. Accordingly, further information about the etiologies of the restrictive spirometric pattern could not be obtained. Thus, instead of the term “restrictive disease”, “restrictive spirometric pattern” is used for the appropriateness of the nomenclature^36^.

## Conclusion

In the current study, we found that driving pressure is the only modifiable intraoperative factor contributing to PPC risk in patients with restrictive spirometric pattern. PPC risk was decreased in relation to lower driving pressure. Only small decrease in driving pressure was significantly associated with lower PPC risk. In contrast, driving pressure was not significantly associated with PPC risk in patients with normal spirometric pattern. Our findings suggest that optimal intraoperative lung protective strategy may differ by baseline lung physiology or preoperative spirometric pattern.

## Supplementary Information

Supplementary Information.

Supplementary Tables.

## Data Availability

The data that support the findings of this study are available on request from the corresponding author. The data are not publicly available due to privacy or ethical restrictions.
