# Supplementary material for: The impact of driving pressure on postoperative pulmonary complication in patients with different respiratory spirometry
Source: Sci Rep. 2022 Dec 3;12:20875. doi: 10.1038/s41598-022-24627-2 (PMC9719554; doi:10.1038/s41598-022-24627-2)
Supplement: Supplementary file 2 — Supplementary Tables. [file 41598_2022_24627_MOESM2_ESM.docx]

**Supplementary Table S1**. Definition of postoperative pulmonary complication from previously published articles.

| **PPC** | **Definition** |
| --- | --- |
| Respiratory failure | When postoperative PaO_2_ <60 mmHg on room air, a ratio of PaO2 to inspired oxygen fraction < 300 or arterial oxyhemoglobin saturation measured with pulse oximetry < 90% and requiring oxygen therapy |
|  |  |
| Respiratory infection | When a patient received antibiotics for a suspected respiratory infection and met at least one of the following criteria: new or changed sputum, new or changed lung opacities, fever, leukocyte count > 12,000/µL |
|  |  |
| Pleural effusion | Chest x-ray demonstrating blunting of the costophrenic angle, loss of the sharp silhouette of the ipsilateral hemidiaphragm in upright position, evidence of displacement of adjacent anatomical structures, or (in supine position) a hazy opacity in one hemithorax with preserved vascular shadows |
|  |  |
| Atelectasis | Lung opacification with a shift of the mediastinum, hilum, or hemidiaphragm toward the affected area, and compensatory over-inflation in the adjacent nonatelectatic lung |
|  |  |
| Bronchospasm | Newly detected expiratory wheezing treated with bronchodilators |

**Supplementary Table S2.** Intraoperative mechanical ventilation variables of patients with or without postoperative pulmonary complications (PPCs) in the whole study cohort.

|  | **Descriptive statistics** | |  | **Univariable analysis** | |
| --- | --- | --- | --- | --- | --- |
|  | Without PPCs (n=594) | With PPCs (n=60) |  | OR (95% CI) | p-value |
| **Mechanical Ventilation parameters** |  |  |  |  |  |
| Static compliance (ml/cmH_2_O) | 38.8 (31.4-48.0) | 34.7 (28.9-46.7) |  | 0.98 (0.95-1.00) | 0.050 |
| Driving pressure (cmH_2_O) | 11.5 (10.0-14.0) | 13.0 (11.0-14.5) |  | 1.11 (1.01-1.21) | 0.026 |
| Tidal volume per kilogram predicted body weight (mL/kg) | 7.0 (6.1-7.9) | 7.2 (6.1-8.2) |  | 1.1 (0.96-1.27) | 0.173 |
| Plateau pressure (cmH_2_O) | 13 (12-15) | 15 (12-16) |  | 1.07 (0.98-1.16) | 0.116 |
| PEEP (cmH_2_O) | 2 (2-3) | 2 (2-3) |  | 0.85 (0.68-1.05) | 0.123 |

Data are presented as median (25th percentile, 75th percentile) or frequency (%). PEEP, positive end-expiratory pressure.
